# Split Tibialis Anterior Tendon Transfer to The Peroneus Brevis or Tertius for the Treatment of Varus Foot Deformities in Children with Static Encephalopathy: A retrospective case series

**DOI:** 10.5435/JAAOSGlobal-D-20-00044

**Published:** 2020-05-04

**Authors:** Brett Lullo, Alexander Nazareth, Susan Rethlefsen, Kenneth D. Illingworth, Oussama Abousamra, Robert M. Kay

**Affiliations:** From the Department of Orthopaedic Surgery, Harbor-UCLA Medical Center, Torrance, CA (Dr. Lullo); Keck School of Medicine, University of Southern California (Dr. Nazareth, Dr. Illingworth, Dr. Abousamra, and Dr. Kay); the Children's Hospital of Los Angeles (Ms. Rethlefsen, Dr. Abousamra, and Dr. Kay); and the Children's Orthopaedic Center, Children's Hospital Los Angeles (Dr. Illingworth), Los Angeles, CA.

## Abstract

**Introduction:**

The study purpose was to determine the safety/efficacy of a split anterior tibialis tendon transfer (SPLATT) to the peroneus tertius or brevis in children with static encephalopathy and varus feet.

**Methods:**

A retrospective review of short- and long-term complications, change in ankle range of motion, strength, and gait kinematics. Predictors of postoperative varus or valgus were examined.

**Results:**

One hundred thirty-three patients were included (average age [SD] 10.3 [3.7]), with an average follow-up of 3.9 (3.4) years. Forefoot/hindfoot eversion range of motion improved (*P* ≤ 0.05), dorsiflexor strength was maintained or improved in 76.9% of patients, and dorsiflexion in swing phase was maintained. Complications occurred in 6 of 133 patients (4.5%) and included 1 transfer failure, 1 wound dehiscence, and four pressure areas from casts. Successful correction was achieved in 77% of patients. Later onset of recurrent varus (14.4%, 10.6% requiring revision surgery) and pes valgus (8.7%, 4.8% requiring revision surgery) occurred. The length of the follow-up predicted the development of the pes valgus (odds ratio 1.28, 95% CI 1.0 to 1.6).

**Discussion:**

SPLATT to the peroneus tertius or brevis is effective, and complications are rare. Subsequent valgus or recurrent varus deformities may occur, possibly requiring repeat surgery.

Children with static encephalopathy are prone to soft-tissue contractures, bony deformities, and limited mobility.^1^ The soft-tissue contractures and bony deformities in static encephalopathy tend to progress with time, although the brain injury is nonprogressive.

Varus foot deformities are present in 10% of diplegic and quadriplegic children and 30% of hemiplegic children referred for a gait analysis^2^ and are also quite common in nonambulatory children. Varus is most commonly caused by an overpull of the tibialis anterior (AT), the tibialis posterior (PT), or both and may be seen in isolation or in conjunction with equinus deformities.^1^ The AT alone is responsible for the varus deformity in approximately one-third of the cases and in combination with the PT in one-third of cases.^3^ Varus limits stability in the stance phase of gait and interferes with prepositioning of the foot for the stance phase.^1^ Pes varus also often causes notable difficulties with brace and shoe wear. Initial treatment is nonsurgical, consisting of braces, stretching splints or casts, passive stretching, and/or botulinum toxin injections. Surgery is indicated if these nonsurgical interventions fail to adequately control the foot position.

Although a whole tendon transfer of the AT is often successful for a correction of forefoot supination associated with idiopathic clubfoot deformity (Knutsen et al, Kuo et al, and Thompson et al),^4-6^ the whole tendon transfer is generally avoided in the neuromuscular cases because of the concern of achieving balanced coronal plane foot positioning in the short- and long-term in the presence of spasticity and/or dystonia. This can be particularly challenging because tone, strength, and movement disorders often change and evolve over time. Split anterior tibialis tendon transfer (SPLATT) is a well-described treatment of varus foot deformities in individuals with neuromuscular disorders.^7,8,9,10,11,12^ The split tendon has classically been transferred to the cuboid^9-11,13^ or base of the fifth metatarsal.^14^ These transfers to bone, which have often been performed by suturing over a plantar bolster or felt and a button, can result in skin callus, tissue necrosis, plantar hypersensitivity, and transfer failure in patients with spasticity.^9,10,13,14^ Some recent authors have reported the use of interference screws or suture anchors in conjunction with SPLATT, although these devices are expensive and have a shorter track record.^13,14,15,16,17^

Although the results of split transfer to the peroneal tendons have been reported for the PT,^18^ they have not previously been reported for the AT. The purpose of this study was to assess the safety and efficacy of SPLATT to the peroneal tendons in children with static encephalopathy and varus feet.

## METHODS

A retrospective case series study was conducted. The study was approved by the Institutional Review Board at Children's Hospital Los Angeles, with a waiver of consent for retrospective use of data collected previously for clinical purposes (Children's Hospital Los Angeles-18-00058). Medical records were reviewed for all patients with static encephalopathy treated with SPLATT procedures at a single tertiary pediatric hospital from 2001 to 2017. Patients were included in the study if they had a varus foot deformity secondary to static encephalopathy and underwent a SPLATT procedure with transfer of the split tendon to either the peroneus tertius or brevis tendon. Patients were excluded from the study if they underwent a whole tendon transfer, a split transfer to a bony insertion site, or they did not undergo a tendon transfer for a varus foot deformity.

### Surgical Indications

Surgical correction of pes varus was indicated in cases of foot deformity interfering with foot stability in stance and clearance in swing, causing pain or inability to tolerate braces or shoes and when nonsurgical interventions had failed to control foot position.

Dynamic electromyography and a computerized gait analysis testing help determine the deforming force, AT, posterior, or both for ambulatory patients.^3^ The muscle(s) with inappropriate timing of activity (out-of-phase, continuous, and prolonged) are candidates for transfer. Dynamic electromyography was used in the subset of subjects who had a gait analysis testing performed. For subjects who did not undergo a computerized gait analysis, the decision of whether to perform a SPLATT procedure was made using careful clinical examination in the clinic and the judgment of the treating physician.

### Surgical Procedure

The procedures were performed under tourniquet control. If SPLATT was undertaken as part of isolated foot and ankle surgery, a nonsterile tourniquet was used, whereas a sterile tourniquet was used for multilevel surgery.

**Figure 1:**
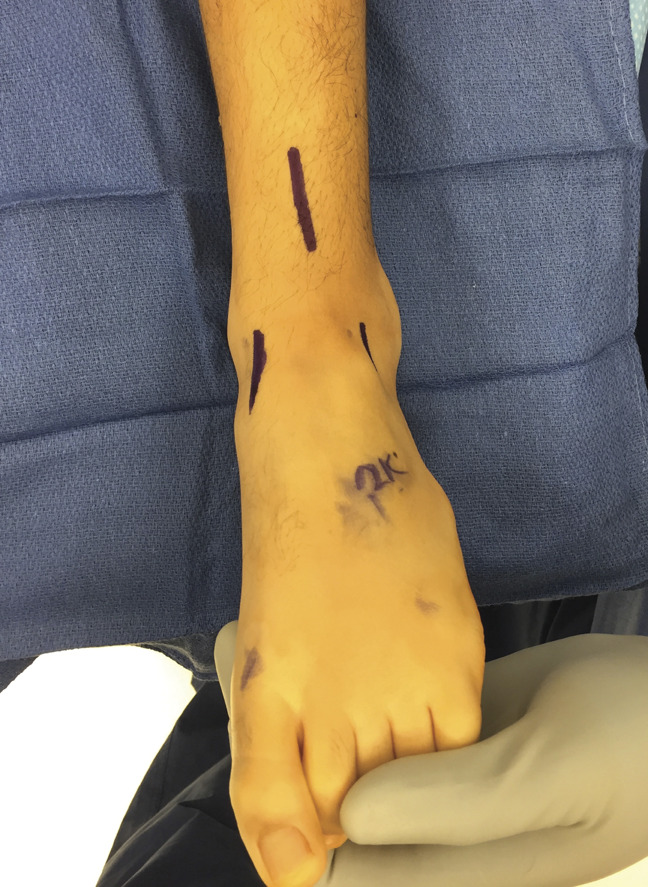
Photograph showing the incision sites for SPLATT to the peroneus tertius or brevis. (Courtesy of Children's Orthopedic Center, Los Angeles, CA.)

Three incisions were made: a 3 to 4 cm oblique incision over the distal course of the anterior tibial tendon, a 4 to 5 cm longitudinal incision lateral to the tibial crest over the anterior lower leg, and a 1.5 to 2 cm oblique incision just distal to the ankle joint, immediately lateral to the toe extensor tendons (Figure 1).

**Figure 2:**
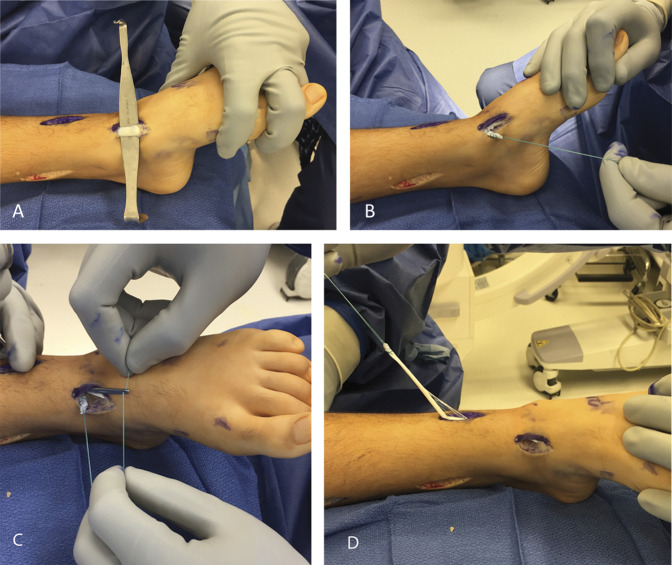
Photograph showing the anterior tibial tendon harvesting and splitting. **A,** Anterior tibialis tendon identified at the distal, medial incision; (**B**) Plantar half of the tendon is harvested and a nonabsorbable 2-0 whip stitch is placed through the distal stump; (**C**) An Ober tendon passer passed through lower leg incision (deep to extensor retinaculum) and the whip stitch retrieved; (**D**) The tendon splits longitudinally as the suture is retrieved. (Courtesy of Children's Orthopaedic Center, Los Angeles, CA.)

The plantar half of the anterior tibial tendon was harvested through the distal, medial incision and a whip stitch was placed through the distal stump. A tendon passer was passed from the lower leg incision (deep to the extensor retinaculum) to retrieve the whip stitch previously placed through the distal stump of the split AT tendon. Because the suture was retrieved, the tendon split in half. This was brought proximally, although with a tendon-to-tendon transfer, obtaining sufficient tendon length was almost never an issue (Figure 2, A–D).

**Figure 3:**
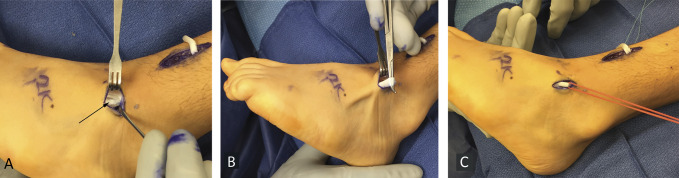
Photograph showing the peroneus tertius identification. **A,** Peroneus tertius located adjacent to fifth toe extensor (black arrow); (**B**) Pulling on peroneus tertius everts foot without moving the fifth toe, and the tendon course is seen distally; (**C**) Vessel loop placed around the tendon. (Courtesy of Children's Orthopaedic Center, Los Angeles, CA.)

The peroneus tertius was located deep to the extensor retinaculum, immediately adjacent to the long extensor to the fifth toe. For accurate tendon identification, tension was exerted the peroneus tertius to (1) make the distal course of the tendon evident, (2) evert the foot, and (3) ensure it did not move the fifth toe. A vessel loop was placed around the peroneus tertius to facilitate later identification (Figure 3, A–C).

If the peroneus tertius was either absent or quite hypotrophic, the dorsal half of the peroneus brevis was used to receive the transfer. In such cases, the peroneus brevis was split longitudinally, left connected distally, and the dorsal half was transected proximally in the hindfoot. A whip stitch was placed in the tendon stump.

**Figure 4:**
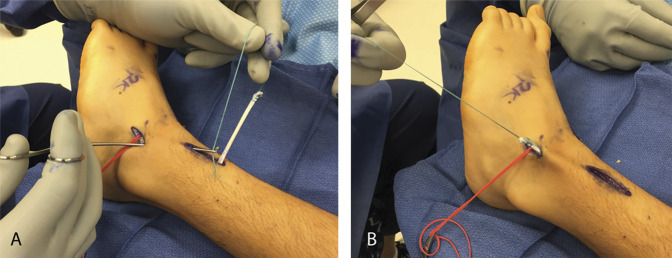
Photograph showing the split tendon transfer. **A** and **B,** Split anterior tibialis tendon retrieved and passed outside the retinaculum to dorsolateral hindfoot adjacent to the peroneus tertius. (Courtesy of Children's Orthopaedic Center, Los Angeles, CA.)

**Figure 5:**
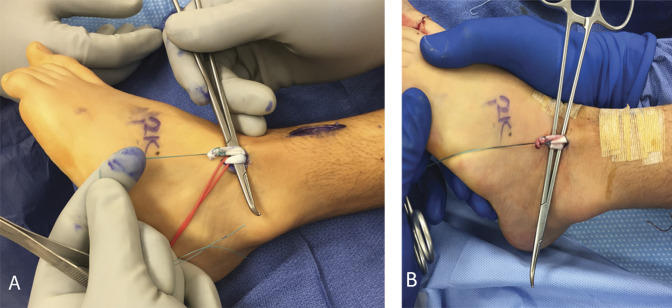
Photograph showing suturing of the transferred tendon. **A**, Side-to-side transfer; **B**, Tendons sutured in a side-to-side fashion with two or three nonabsorbable 2-0 figure 8 sutures while the ankle is held in eversion, with more tension placed on the transferred portion of the tendon than the tension in the medial (native) half of the tendon. (Courtesy of Children's Orthopaedic Center, Los Angeles, CA.)

The split anterior tibial tendon was passed outside the retinaculum to the dorsolateral hindfoot, adjacent to the peroneus tertius (Figure 4, A and B). All incisions, except that through which the tendon transfer was to be completed, were closed in a standard fashion. After all other incisions were closed, the tendon transfer was completed in a side-to-side fashion with two or three nonabsorbable 3-0 sutures, with the ankle held in neutral dorsiflexion and the foot in eversion (Figure 5, A and B). The peroneus tertius was left attached distally but did not need to be transected proximal to the transfer insertion site. The final incision was closed, and the foot was maintained in this position until short leg casting was completed. If only soft-tissue procedures were performed, the child was allowed to weightbear as tolerated immediately. Casting was for 6 weeks and part-time brace wear (for at least 6 to 8 hours daily) was used until at least 6 months postoperatively.

### Data Collection

Medical records were reviewed from the date of preoperative gait analysis (if done) or the date of surgery through the most recent follow-up appointment. Demographic parameters collected from the records included age at surgery, sex, height and weight at surgery, underlying neuromuscular diagnosis, the Gross Motor Function Classification System (GMFCS) level,^19,20^ concomitant surgeries, and the length of the follow-up.

In addition to demographics, details about postoperative complications related to the SPLATT were collected and rated according to the Modified Clavien-Dindo (MCD) complication rating system.^21-23^ Postoperative recurrence of pes varus, development of valgus foot deformities, need for, and the type of revision surgeries were also recorded.

Preoperative and postoperative gait analysis data were available for a subset of 40 patients, along with clinical examination at both these time points by the motion analysis laboratory staff. Clinical examination measures recorded from gait analysis assessments included passive hindfoot and forefoot eversion range of motion and ankle dorsiflexion strength. Computerized gait analysis was performed using a Vicon motion capture system (Vicon Motion Systems). Fifteen to 19 retroreflective markers were placed on the subject's lower body according to the Plug-in-Gait Model and kinematic data were collected at 120 Hz. The subjects walked barefoot along a 15-m walkway at a self-selected speed, using assistive devices as necessary. Data from a representative stride were used for data analysis. Gait analysis parameters recorded included mean and maximum ankle dorsiflexion during the swing phase and the timing of the maximum ankle dorsiflexion in the swing phase. The reason for postoperative testing varied among subjects. Many were seen at approximately 1 year postoperatively to assess the outcome of surgery and determine the therapy and bracing treatment needs. Others were seen for repeat preoperative testing to assess the need for further surgery at other joints or repeat surgery at the foot/ankle level.

Records of patients with more than 1-year of follow-up were reviewed for recurrence of pes varus and development of pes valgus. These were considered present if noted and recorded by the surgeon in any follow-up clinic note or if noted and recorded in the postoperative gait analysis report.

One hundred thirty-three patients fit the eligibility criteria and were included in the analysis. A SPLATT to the peroneus tertius was performed in 103 of 133 patients (77.4%), and 30 of 133 (22.6%) underwent a transfer to the peroneus brevis tendon. There were 66 men (49.6%) and 67 women (50.4%) in the group. The average age at surgery was 10.3 (3.7) years (range, 4.1 to 20.1). The average body mass index at surgery was 18.2 (4.1) kg/m^2^ (range, 12.6 to 37.4). There was unilateral involvement in 41 of 133 subjects (31%) and bilateral involvement in 92 of 133 subjects (69%). The average length of the follow-up was 3.9 (3.4) years (range, 0.1 to 13.8). Ninety-three percent of subjects had diagnoses of cerebral palsy, and the remaining subjects had other types of static encephalopathy (chronic traumatic brain injury or stroke, Angelman syndrome, Cri-du-chat, acute disseminated encephalitis, and hydrocephalus). The distribution of the GMFCS levels among subjects with CP was level I (32/124, 26%), level II (36/124, 29%), level III (15/124, 12%), level IV (20/124, 16%), and level V (21/124, 17%).

### Statistical Analysis

To minimize sampling bias, one limb was analyzed per subject. Right strides (selected by coin toss) were analyzed for participants with bilateral SPLATT, whereas the surgical side was used in unilateral cases. The paired Student *t*-test was used to determine notable differences between preoperative and postoperative range of motion, strength, and gait measurements. Two sample *t*-tests, Fisher exact test, and logistic regression were used to determine the predictors of postoperative recurrent pes varus and development of pes valgus. Statistical significance was determined with an alpha value of 0.05.

## RESULTS

Most transfers were done as part of a single-event multilevel surgery. Concomitant foot and ankle surgeries included gastrocnemius recession (39.1% of subjects), tendo-Achilles lengthening (23.6%), calcaneal and midfoot osteotomies (30% each), posterior tibialis tendon lengthening (54.1%), and split posterior tibialis tendon transfer (29.3%).

Complications occurred in 6 of 133 patients (4.5%). There was one case of transfer failure requiring revision surgery (MCD grade III) and one case of a small wound dehiscence (MCD grade II), which was treated successfully with antibiotics (1/133, 0.8% each). There were 4 cases (4/133, 3%) of pressure areas from casts requiring no additional medical intervention (MCD grade 1).

Among the 104 patients with greater than a 1-year follow-up, recurrent pes varus occurred in 15 of 104 limbs (14.4%), of which 11 of 104 requiring revision surgery (10.6%). Pes valgus developed over time in 9 of 104 limbs (8.7%), of which 5 of 104 requiring revision surgery (4.8%). Only two subjects with less than 2 years of follow-up had developed recurrent pes varus, and none had developed pes valgus by that time. Most recurrent varus and all cases of pes valgus occurred in patients with more than 2 years of follow-up, with increasing frequency and increasing time (Table 1).

**Table 1:** Frequency of Recurrent Varus and Development of Pes Valgus by the Length of the Follow-up

Length of the follow-up group (yr)	Recurrent Varus		Development of Valgus	
	Count	%	Count	%
0-1	0	0	0	0
1-2	2	9	0	0
2-5	6	15	2	5
5+	7	16	7	16

Postoperative hindfoot eversion range of motion improved by an average (SD) of 3 (9)° (*P* = 0.05), and forefoot eversion improved 6 (13)° (*P* = 0.01). Ankle dorsiflexion strength was maintained or improved postoperatively in 20 of 26 patients (76.9%) in whom it was tested (Table 2), irrespective of the concomitant gastrocnemius recession or tendo-Achilles lengthening (*P* = 0.21).

**Table 2:** Change in Ankle Dorsiflexion Strength Based on Manual Muscle Testing

	All Patients (n = 26)		Patients With GR or TAL (n = 13)		Patients Without GR or TAL (n = 13)	
	Count	%	Count	%	Count	%
−2 grades	1	3.8	0	0.0	1	7.7
−1 grade	5	19.2	1	7.7	4	30.8
0 grades	3	11.5	3	23.1	0	0.0
1 grade	15	57.7	8	61.5	7	53.8
2 grades	2	7.7	1	7.7	1	7.7

GR = gastrocnemius recession, TAL = tendo-Achilles lengthening

Among patients who did not undergo simultaneous gastrocnemius recession or tendo-Achilles lengthening, mean and maximum ankle dorsiflexion in the swing phase of gait were not changed significantly preoperatively to postoperatively. Timing of peak dorsiflexion in swing was also unchanged (Table 3).

**Table 3:** Swing Phase Gait Kinematic Parameters (Patients Without Concomitant Gastrocnemius Recession or Tendo-Achilles Lengthening Only, N = 18)

	Presurgery		Postsurgery		Change		
	Mean (SD)	Range	Mean	Range	Mean	Range	*P* Value
Mean DF (deg)	−12 (14)	−44 to 8	−8 (12)	−38 to 10	3 (15)	−29 to 43	0.38
Max DF (deg)	−5 (12)	−31 to 15	−3 (13)	−36 to 16	2 (15)	−28 to 36	0.54
DF timing (% swing)	56 (43)	0 to 100	62 (34)	0 to 100	7 (53)	−100 to 100	0.60

DF = dorsiflexion

Potential predictors of recurrent varus or development of valgus considered for analysis included age at surgery, body mass index, sex, length of follow-up, the GMFCS level, transfer site, concomitant calcaneal osteotomy, PT tendon lengthening, or split transfer. None of these factors were predictive of recurrent varus (*P* > 0.10). Only the length of follow-up was markedly related to the development of pes valgus (odds ratio 1.28, 95% CI 1.0 to 1.6).

## DISCUSSION

Split transfer to the peroneus brevis for pes varus has been described for the PT, but split transfer to peroneal tendons has not been described for the AT. Split transfer of the AT tendon is traditionally done to bone and can be associated with complications such as skin callus and tissue necrosis from button fixation in patients with spasticity. The purpose of this study was to assess the safety and efficacy of SPLATT to the peroneal tendons in children with static encephalopathy and varus feet.

SPLATT to the peroneus tertius or brevis is a safe procedure. In the current study, there was one MCD grade III complication—a failure of the transferred tendon requiring revision surgery, one MCD grade II perioperative complication—a small wound dehiscence which did not require revision surgery, and four MCD grade I cast pressure areas. These complications are common with any tendon transfer procedure, less frequent in our series than the previously described techniques. In a study by Vogt et al reviewing the results after 132 SPLATT procedures to the cuboid, five patients (4%) had a rupture or necrosis of the transferred tendon, five patients (4%) had wound complications, and two patients (1.5%) developed a complex regional pain syndrome.^12^ In a study of 47 adult patients with spastic equinovarus feet who underwent a SPLATT to the cuboid via bone tunnel using interference screw fixation, Hosalkar et al. compared the traditional dorsoplantar routing of the split tendon into the cuboid with a lateromedial routing; there were three fixation-related complications (3/17 = 18%) and one wound healing complication (1/17 = 6%) in the dorsoplantar group and zero complications of either screw pullout or wound healing complications in the lateromedial group.^13^ Gasse et al.^14^ performed the SPLATT on 22 patients with spastic equinovarus foot deformities with fixation of the split tendon to the base of the fifth metatarsal via bone anchor; they argued that this would be less traumatic to the bone than a bone tunnel and less traumatic to the sole of the foot than transplantar fixation. At a minimum follow-up of 2 years, they reported 100% satisfaction but noted one detachment of the bone anchor (1/22 = 4.5%) at 6 months post-op.

Avoiding transfer of the split tendon to bone may decrease the occurrence of these complications. Kling et al.^18^ used a split PT tendon transfer to the peroneus brevis in 31 children with CP (average age 8.0 years) and spasticity of the PT tendon throughout the gait cycle. At an average of 8.0 years of follow-up, there were 34 patients with excellent or good results (91.9%) and two complications (6%), including skin flap necrosis and one superficial wound infection.

A common technical issue with SPLATT to bone is that it is often challenging to obtain sufficient tendon length to complete the transfer optimally. Obtaining sufficient tendon length is never an issue when performing SPLATT to the peroneus tertius, and rarely an issue when performing a SPLATT to the peroneus brevis. The key to obtaining sufficient length when using the peroneus brevis is to transect the dorsal half of the split tendon as proximally as possible in the hindfoot.

Patients in our study had notable improvements in passive forefoot and hindfoot eversion range of motion postoperatively. However, because other foot surgeries were performed simultaneously with SPLATT to the peroneal in many subjects, passive range improvements may not be attributable solely to SPLATT. Ankle dorsiflexion strength was maintained or improved by at least one muscle grade in most patients. Timing and magnitude of maximum dorsiflexion in the swing phase of gait was unchanged postoperatively among our patients who did not have simultaneous triceps surae lengthening. These findings suggest that a SPLATT to the peroneal tendons is effective in correcting varus without compromising dorsiflexor strength or function for foot clearance in swing during gait. This is very important because the dogma stating that one grade of muscle strength is lost when tendon transfers are performed was contradicted by the results in the current study. There are likely at least a few reasons for this: (1) the SPLATT was passed outside the retinaculum to the foot, to allow a direct line of pull, without tethering by the retinaculum, (2) tensioning the lateral band tighter than the medial (native) band of the AT, and (3) maintaining the ankle in neutral dorsiflexion and the hindfoot in neutral to eversion after the transfer was completed and while applying the postoperative cast.

The results of SPLATT have overall been positive in the treatment of varus foot deformities in individuals with neuromuscular disorders. In a study of 73 feet (69 patients with average age 46.5 years) that underwent a SPLATT to the cuboid for spastic equinovarus foot deformities, Vogt reported notable improvement in patient autonomy at an average follow-up of 44 months, including improved ability to ambulate independently, decreased need to wear orthopaedic shoes and orthoses, and increased ability to wear normal shoes.^9^ Three patients (4%) required revision surgery for recurrence of deformity. Limpaphayom et al. performed a SPLATT to the cuboid on 68 equinovarus feet in 45 ambulatory children with CP (average age 8.1 years); they reported excellent or good outcomes in 85% of feet and improved the GMFCS levels in 34 children (75.6%).^11^ Ten percent of children had a poor outcome, which was predicted by a pretreatment GMFCS of 3 to 4. By contrast, our current results show no predictive value of the GMFCS level for recurrent varus or the development of pes valgus.

Although functional gait data were only available for a subset of our patients, our collection of objective parameters preoperatively and postoperatively is still a strength of our study. Most other studies of patients with spastic varus foot deformity who underwent SPLATT rely on subjective measures of success, such as patient satisfaction, or group patients into broad-based, ill-defined outcome categories. These objective, detailed measurements allowed us to more precisely assess preoperative and postoperative change and directly quantify the improvement in our patients.

Overall, a SPLATT to the peroneal tendons led to successful deformity correction in 77% of patients. However, an unintended result of late-onset valgus or recurrent pes varus occurred in approximately 23% of cases, most often more than 2 years postoperatively, a portion of which required revision surgery. We were unable to determine the predictors of recurrent pes varus. Recurrent or residual varus deformity may occur for several reasons after a SPLATT.^1^ First, the AT may be improperly identified as the cause of varus instead of the PT. This should be avoidable with preoperative gait analysis using computerized motion analysis and dynamic electromyography, as was the case for many, but not all, of the patients in our study. Second, a rigid deformity could go unrecognized and untreated. Indeed, 6 of 11 subjects with recurrent varus requiring repeat surgery went on to have calcaneal and/or midfoot osteotomy. In addition, whether a child with CP has had surgery, the pattern of muscle spasticity, tightness, and/or dystonia may change and evolve over time, which may also cause recurrence of deformity or even a reversal of deformity (eg, from varus to valgus). Our data show that the length of the follow-up was predictive of development of pes valgus after SPLATT to tendon, a finding which accords with the current literature. Development of a pes valgus deformity is known to occur over time and is part of the natural history of gait in children with CP.^2,24^ This is likely secondary to physiologic changes in muscle balances as a child with static encephalopathy grows.^1^

One of the limitations of our study is the average length of our follow-up (3.9 years), which may limit our ability to accurately account for all long-term complications and assess changes in the functional outcomes over the long term as children grow. Another limitation of our study is the relatively low number of patients for whom we have computerized motion analysis data, along with the thorough static examination data obtained at such visits, including objective range of motion, strength, and computerized gait data. Exhaustive range of motion, strength, and gait data were only collected on the subset of patients at the time of computerized motion analysis. This may limit our ability to assess true differences within the entire population but still serves as a representative sample. Last, we were unable to include objective preoperative and postoperative measures of foot alignment to assess the outcome. In future, the addition of pedobarography, foot alignment assessments such as the Foot Posture Index, and even the use of a multisegmental kinematic foot model would be useful to assess the magnitude of deformity and surgical outcome.

SPLATT to the peroneus tertius or brevis is a safe and effective for treatment of varus foot deformities in children with static encephalopathy. Complications are rare, and no major wound issues were seen. Despite successful correction in 77% of cases, late-onset valgus and recurrent varus deformities occur in some cases, which may require repeat surgical intervention. This study presents a new surgical SPLATT technique that may be as effective as the current standard of care and may avoid wound complications associated with transfer to bone.
